# Taurine/chenodeoxycholic acid ratio as a potential serum biomarker for low vitamin B_12_ levels in humans

**DOI:** 10.1017/S0007114524002022

**Published:** 2024-09-28

**Authors:** Madhu Baghel, Sting L. Shi, Himani Patel, Vidya Velagapudi, Abdullah Mahmood Ali, Vijay K. Yadav

**Affiliations:** 1 National Institute of Immunology, New Delhi, India; 2 Systems Biology of Aging laboratory, Department of Genetics and Development, Columbia University, New York, NY, USA; 3 Institute for Molecular Medicine Finland FIMM, University of Helsinki, Helsinki, Finland; 4 Department of Medicine, Columbia University Irving Medical Center, New York, NY, USA; 5 Department of Pathology, Immunology and Laboratory Medicine, Rutgers University, Newark, NJ, USA; 6 Center for Cell Signaling, Rutgers New Jersey Medical School, Newark, NJ, USA; 7 Center for Immunity and Inflammation, Rutgers University, Newark, NJ, USA

**Keywords:** Vitamin B_12_, Taurine, Metabolism, Ageing, Metabolomics, Biomarkers

## Abstract

Deficiency of vitamin B_12_ (B_12_ or cobalamin), an essential water-soluble vitamin, leads to neurological damage, which can be irreversible and anaemia, and is sometimes associated with chronic disorders such as osteoporosis and cardiovascular diseases. Clinical tests to detect B_12_ deficiency lack specificity and sensitivity. Delays in detecting B_12_ deficiency pose a major threat because the progressive decline in organ functions may go unnoticed until the damage is advanced or irreversible. Here, using targeted unbiased metabolomic profiling in the sera of subjects with low B_12_ levels *v* control individuals, we set out to identify biomarker(s) of B_12_ insufficiency. Metabolomic profiling identified seventy-seven metabolites, and partial least squares discriminant analysis and hierarchical clustering analysis showed a differential abundance of taurine, xanthine, hypoxanthine, chenodeoxycholic acid, neopterin and glycocholic acid in subjects with low B_12_ levels. Random forest multivariate analysis identified a taurine/chenodeoxycholic acid ratio, with an AUC score of 1, to be the best biomarker to predict low B_12_ levels. Mechanistic studies using a mouse model of B_12_ deficiency showed that B_12_ deficiency reshaped the transcriptomic and metabolomic landscape of the cell, identifying a downregulation of methionine, taurine, urea cycle and nucleotide metabolism and an upregulation of Krebs cycle. Thus, we propose taurine/chenodeoxycholic acid ratio in serum as a potential biomarker of low B_12_ levels in humans and elucidate using a mouse model of cellular metabolic pathways regulated by B_12_ deficiency.

Vitamin B_12_ (B_12_ or cobalamin) is an essential water-soluble vitamin derived from animal-based diets which function to regulate two immediate cellular processes in one-carbon metabolism and Krebs cycle^(1–5)^. The absorption of dietary B_12_ requires gastric intrinsic factor (GIF), a stomach-specific protein^(4)^. Gif binds to B_12_ in the small intestine, forming the GIF-B_12_ complex, which is then endocytosed by the ileal epithelial cells, and B_12_ is released into the bloodstream^(4)^. In the bloodstream, B_12_ binds primarily to the proteins haptocorrin and transcobalamin. Transcobalamin transports B_12_ to peripheral organs, including the liver, which is the primary storage and recycling organ for B_12_ in mammals^(6)^. Once acquired, humans, for instance, can recycle B_12_ to maintain B_12_-dependent cellular processes for up to a decade, if the intestinal B_12_ absorptive processes are intact and unimpaired as well as daily dietary requirements are met to maintain B_12_ storage in the body^(2)^. In the cells, B_12_ derivatives function as cofactors for only two known enzymes: methylmalonyl-CoA mutase and methionine synthase and affect downstream metabolic pathways such as Krebs cycle and one-carbon metabolism^(1,7)^. In humans, decreased production of functional GIF protein or non-consumption of animal products causes B_12_ deficiency and results in various abnormalities, such as anaemia and cognitive defects and often other degenerative diseases^(8–11)^.

In clinical practice, the diagnosis of B_12_ deficiency is typically established by the measurement of serum cobalamin (Cbl) levels^(12)^. Although B_12_ deficiency can be reflected by decreased holotranscobalamin (holoTC), methylmalonic acid (MMA) and homocysteine (HC) levels, these tests are not routinely used unless the initial Cbl levels are equivocal because methylmalonic acid and homocysteine can be elevated in conditions independent of B_12_ levels^(13–17)^. Despite the importance of B_12_ and its association with many physiological functions, several issues remain unresolved in the diagnosis of B_12_ deficiency, leading to poor diagnosis and irreversible consequences on the body^(18,19)^. First, B_12_ is a very stable molecule because 95–97 % of B_12_ is stored in the liver, and although its serum levels reflect tissue levels of B_12_ when the levels are very low, they may not accurately reflect functional levels in some situations^(20)^. For instance, individuals with deficient or absent HC have serum cobalamin values in the deficient range but show no clinical sign of cobalamin deficiency^(21)^. Second, the cost of measurement of B_12_ in patient samples, despite being not able to accurately predict a B_12_-deficient state, remains high and therefore is not the first line of measurement; clinicians measure B_12_ only when a patient presents signs of B_12_ deficiency such as anaemia to confirm a deficient state^(22–25)^. These facts necessitate the need to identify molecules regulated, indirectly or directly, by B_12_, which can provide a functional readout of low B_12_ levels in humans.

We recently created a transgenic mouse model of B_12_ deficiency by deleting the gene essential for B_12_ absorption from the gut, *Gif*, to understand the molecular consequences of B_12_ deficiency. These studies led to the identification that B_12_ stored in the liver regulates the production of taurine. Taurine is a semi-essential micronutrient that has recently been shown to be a driver of ageing as its supplementation increases healthy lifespan in diverse species from worms to mice, and low taurine levels are associated with poor health in aged humans^(26)^. In the animal models, taurine has been documented to play an important role in B_12_ mode of action and regulates growth and bone mass as the reversal of taurine deficiency through daily oral taurine administration was shown to prevent the consequences of B_12_ deficiency on growth and bone mass^(27)^. More importantly, the targeted metabolomics analysis of liver tissue collected from control and B_12_-deficient mice showed changes in a multitude of metabolites besides taurine that are secreted from cells and could be detected in the serum^(27)^. These studies suggested a plausible and testable hypothesis that certain metabolites or sets of metabolites may exist which could serve as a readout of, difficult to detect, low B_12_ state in humans.

The present study was initiated to test the above hypothesis by performing a metabolomic analysis on serum samples collected from subjects with normal and low B_12_ levels to identify which factor(s) could serve as a biomarker of low B_12_ state. Results showed that serum levels of certain metabolites such as taurine, xanthine and hypoxanthine were dramatically lower in the subjects with low B_12_ levels. Using various downstream analyses, we suggest that taurine in conjugation with chenodeoxycholic acid can serve as a biomarker of low B_12_ state in humans. Furthermore, using mouse B_12_-deficient tissues, we elucidate how despite only being needed for two known enzyme functions, B_12_ deficiency alters the metabolic and transcriptomic landscape in the cells, which will facilitate advances in further understanding the biology of B_12_.

## Results

### Study population, sample classification, acquisition, pre-processing and normalisation of metabolomic data

**Fig. 1.**
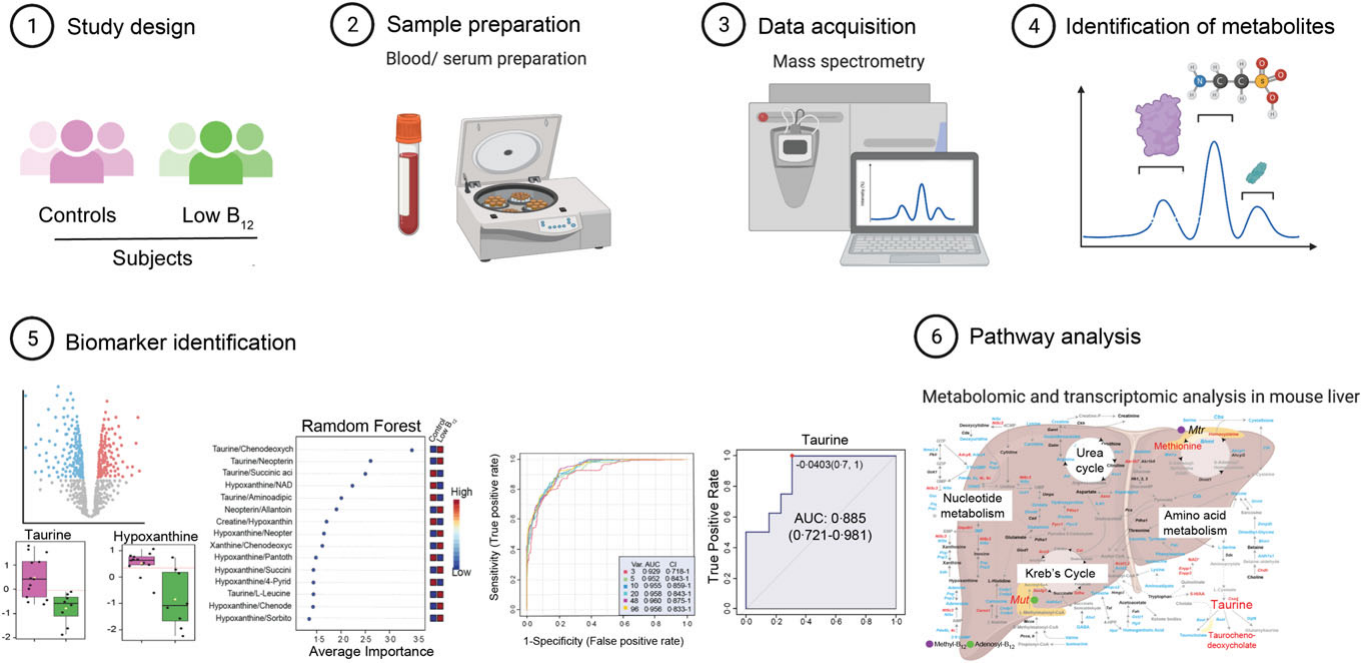
Study population, sample classification, acquisition, pre-processing and normalisation of metabolomic data. Schematic diagram illustrating the steps for metabolomic analysis of serum samples from low B_12_ subjects (B_12_ levels <150 pmol/l) *v* the healthy control group. (1) In this study, eight and thirteen subjects were grouped in low B_12_ and control groups (age- and gender-matched), respectively, (2) blood samples were collected and processed, (3) metabolomics data was acquired from serum samples using ACQUITY UPLC-MS/MS system (Waters Corporation, Milford, MA, USA), data was pre-processed and analysed using MetaboAnalyst 5·0 to identify (4) differentially expressed metabolites between two study groups, (5) serum metabolic biomarker for low B_12_ levels followed by (6) pathway analysis.

A schematic diagram illustrating the different steps of this study is presented in Fig. 1. The samples utilised in this study are a subset from the Kuopio Ischaemic Heart Disease Risk Factor (KIHD) study aimed at identifying the risk factors for CHD, atherosclerosis and other related conditions in the Eastern Finnish population^(28)^. Sera were classified in accordance with internationally established criterion into control subjects (*n* 13) with B_12_ levels >250 pmol/l and into subjects (*n* 8) with low B_12_ levels <150 pmol/l^(1,12,18,29)^. Clinical characteristics of subjects in the control and low B_12_ group are shown in Table 1. Samples were randomised before metabolite extraction and quantified using a ACQUITY UPLC-MS/MS system. Ninety-four metabolites could be detected in the sera, out of which seventy-seven that passed quality control were selected for further downstream analysis (online Supplementary Fig. S1). Imputation of one missing value with the minimum value in that cohort was done, and data were pre-processed by generalised log transformation (glog) and auto-scaling of metabolite concentration peaks in each sample to represent uniform distribution.

**Table 1.** Clinical characteristics of controls and subjects with low B_12_ levels. All values are mean ± sem

Characteristic	Controls		Low B_12_	
	Mean	sem	Mean	sem
Participants (*n*)	13		8	
Age (years)	55·78	0·95	55·18	0·68
Sex	Male		Male	
Energy intake (KJ/4 days)	9634·50	689·36	11 827·98	796·06
Vitamin B_12_ (pmol/l)	412·92	34·48	100·25	12·62
Homocysteine (μmol/l)	10·58	0·84 (5)	16·67	0·14 (3)
Vitamin A (ng/ml)	534·56	12·13	672·93	53·60
Ascorbic acid (mg/l)	5·35	0·78	11·78	1·15
Folate (nmol/l)	7·13	0·61	11·21	1·83
BMI (kg/m^2^)	25·36	0·64	29·27	1·02
Systolic blood pressure (mmHg)	142·10	5·14	134·25	4·50
Diastolic blood pressure (mmHg)	92·05	2·99	91·29	4·49
Serum glucose (mmol/l)	4·43	0·07	4·56	0·15
Serum insulin (mU/l)	8·72	0·83	13·63	1·68
Albumin (g/l)	41·23	0·93	44·13	1·04
Serum total cholesterol (mmol/l)	5·36	0·15	5·82	0·30
LDL-cholesterol (mmol/l)	3·74	0·18	3·81	0·26
HDL-cholesterol (mmol/l)	1·36	0·08	1·23	0·06
VLDL-cholesterol (mmol/l)	0·32	0·07	0·79	0·16
Triglycerides (mmol/l)	0·84	0·07	2·02	0·54
Testosterone (nmol/l)	22·94	1·90	20·70	1·52
Na (mmol/l)	140·92	0·35	141·13	0·71
K (mmol/l)	3·99	0·08	3·88	0·09
Ca (mmol/l)	1 19	0·15	1·21	0·02
Alphatocopherol (μmol/l)	18·17	0·97	21·87	2·12
Uric acid (mmol/l)	0·33	0·01	0·36	0·01
Creatinine (μmol/l)	91·50	3·5	88·50	2·91
Creatinine clearance (ml/s)	1·66	0·13	1·64	0·06
Glutathione peroxidase (mU/gHb)	1·73	0·17	1·70	0·12
Ferritin (μg/l)	167·15	27·12	194·75	63·02
C-reactive protein (mg/l)	1·40	0·23	2·07	0·58

### Identification of differentially expressed serum metabolites in low B_12_ state

**Fig. 2.**
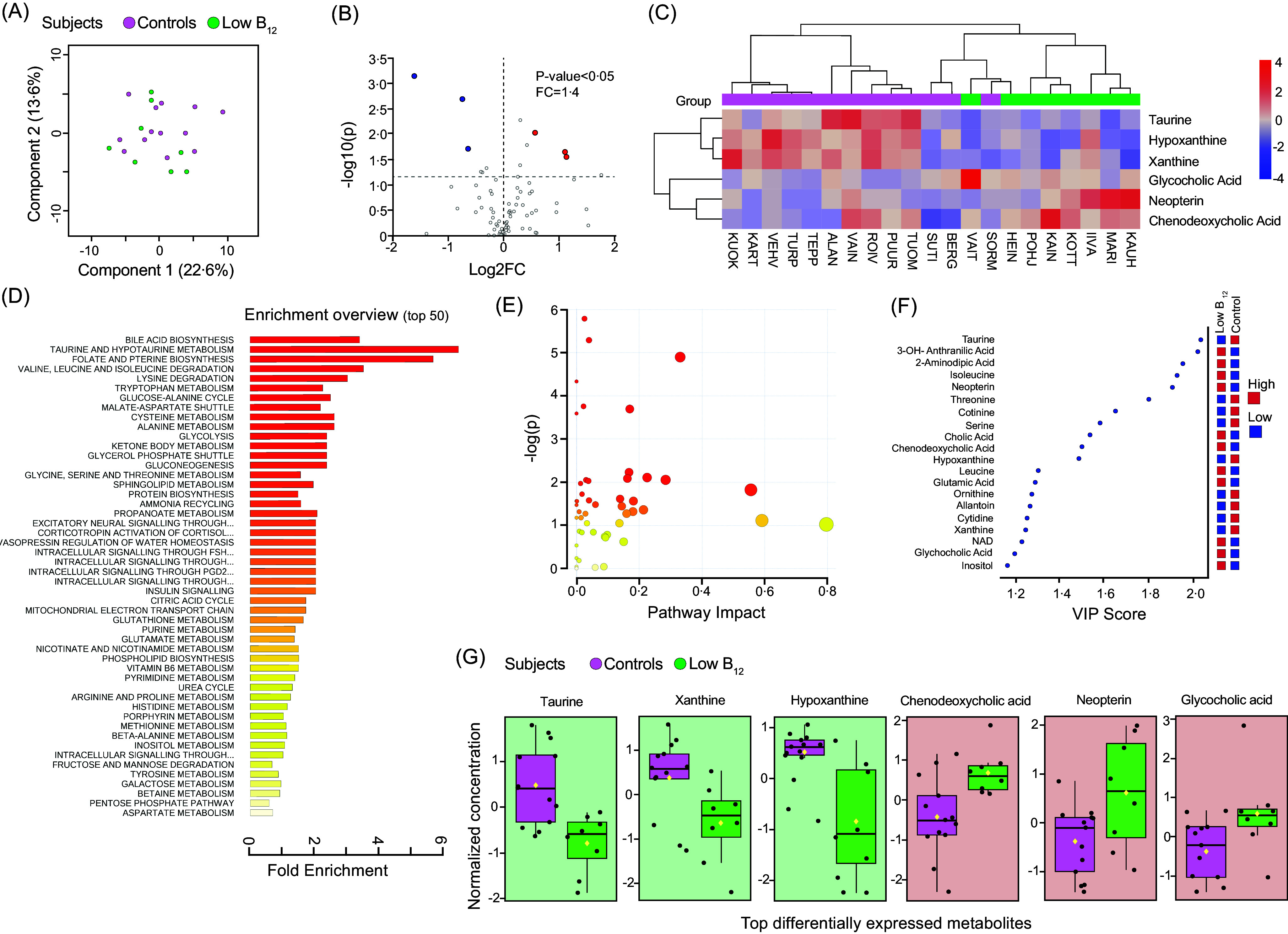
Identification of differentially expressed serum metabolites in low B_12_ subjects. (a) Unsupervised multivariate principal component analysis plot showing the spread of control (pink dots) *v* low B_12_ (green dots) cohort based on the serum metabolic profile. The horizontal and vertical coordinates are the first and second principal components, respectively. Each dot represents a sample. (b) Volcano plot showing six (blue and red dots) most significant differentially expressed metabolites between the low B_12_ subjects *v* controls, with a *P*-value < 0·05 and a log2 fold change ± 0·5. X-axis corresponds to log2(Fold Change) and Y-axis to −log10(*P*-value). (c) Hierarchical clustering analysis sorted the control (pink) *v* low B_12_ (green) group based on the differential abundance of six metabolites (taurine, hypoxanthine, xanthine, glycocholic acid, neopterin and chenodeoxycholic acid). Relative abundance scored from 4 (highest, red colour) to –4 (lowest, blue). (d) Metabolite set enrichment analysis plot with top fifty enriched metabolic pathways (vertical axis) to which the seventy-seven identified metabolites belong. The pathways are arranged in descending order of fold enrichment score (horizontal axis) where the highest is 6 (red colour) and lowest is 0 (yellow colour) (e) Metabolomic pathway analysis plot showing most enriched pathways with significance (–log*P*) values for each of the pathway as dots of red (high significance) or yellow (low significance). X-axis corresponds to pathway impact and Y-axis to –log*P* values. The size of the dot represents its impact value. (f) VIP score plot from PLS-DA analysis showing the top twenty differentially expressed metabolites in serum of control *v* low B_12_ group scored from 1 to 2. Relative abundance is depicted with red (highest) and green (lowest) colour. (g) Box plots showing normalised concentrations of individual metabolites following univariate analysis: taurine (*P* = 0·002), xanthine (*P* = 0·019) and hypoxanthine (*P* = 0·000), chenodeoxycholic acid (*P* = 0·063), neopterin (*P* = 0·023) and glycocholic acid (*P* = 0·027) in the sera of control (red) *v* low B_12_ (green) subjects.

We first performed a principal component analysis, an unsupervised multivariate analysis, to group/classify samples without any consideration of prior classification to detect any outliers in the two cohorts. The principal component 1 (PC1) accounted for 22·6 % of the variance and PC2 accounted for 13·6 % of the variance (Fig. 2(a)). To identify the differential concentration of each metabolite between the control and low B_12_ groups, we calculated the mean fold change and performed *t*-tests to compare the mean of each metabolite. A metabolite was considered significantly different between each group when the value of *P* ≤ 0·05 and log2 fold change ±0·5. In the volcano plot, the three blue dots in the upper left and three red dots in upper right quadrants represent the most significantly altered metabolites in low B_12_ subjects compared with that in controls (Fig. 2(b)). A hierarchical clustering analysis of the metabolomic data using the top three downregulated and top three upregulated metabolites showed well-defined clustering of thirteen healthy subjects (pink, left cluster) *v* eight subjects with low B_12_ levels (green, right cluster) (Fig. 2(c)). The control group showed a high abundance (shades of red colour) of taurine, hypoxanthine and xanthine compared to the low B_12_ group, whereas the abundance of glycocholic acid, neopterin and chenodeoxycholic acid was significantly higher in the low B_12_ group as compared with healthy controls (Fig. 2(c)). Following the identification of differentially expressed metabolites (DEM), we did metabolite set enrichment analysis and metabolomic pathway analysis to determine the metabolic pathways that are associated with differences in the abundance of identified metabolites and perturbations which are associated with the low B_12_ state. The metabolite set enrichment analysis classified the seventy-seven DEM into fifty different metabolic pathways (Fig. 2(d)) that include divergent cellular metabolism pathways such as bile acid biosynthesis, amino acid biosynthesis, glucose metabolism and nucleic acid synthesis, which are listed in the order of descending fold enrichment (Fig. 2(d)). Out of the fifty listed pathways, the taurine and hypotaurine metabolism pathway was the most enriched pathway with the highest fold enrichment value (–log*P* value ∼6). Metabolomic pathway analysis results revealed that the taurine and hypotaurine metabolism pathway had the highest pathway impact value between the controls and subjects with low B_12_ levels, further validating the importance of this pathway (Fig. 2(e)).

Once we identified the most significant DEM and major pathways to which these DEM belonged to, we wanted to check the consistency of identified DEM as most discriminant variables for classifying healthy controls *v* subjects with low B_12_ levels. For this purpose, we performed a partial least squares discriminant analysis (PLS-DA) analysis that helps in highlighting whether a metabolite is upregulated or downregulated in a group/sample by creating a latent structure and the values of variable importance projection score, which represent the importance of the metabolite in the PLS-DA model (Fig. 2(f)). The variable importance projection score plot (threshold of >1·0) revealed that taurine had the maximum score with low abundance in subjects with low B_12_ levels *v* controls (Fig. 2(f)). The other metabolites that were identified in volcano plot i.e. xanthine, hypoxanthine, chenodeoxycholic acid, neopterin and glycocholic acid also came up in PLS-DA plot, suggesting the consistency of these metabolites as important DEM in controls *v* subjects with low B_12_ levels. Further, we performed univariate analysis (*t*-test) on individual DEM to determine the significant difference in the abundance of each metabolite between the two groups. Based on the analysis, the abundance of taurine (*P* = 0·002), xanthine (*P* = 0·019) and hypoxanthine (*P* = 0·000) was significantly lower, whereas the levels of chenodeoxycholic acid (*P* = 0·063), neopterin (*P* = 0·023) and glycocholic acid (*P* = 0·027) were significantly higher in sera of subjects with low B_12_ levels (green bars) compared with healthy controls (pink bars) (Fig. 2(g)).

Metabolites that belong to the same pathway tend to work in coherence. To this end, we subjected the metabolite data to Pearson’s correlation matrix analysis to reveal any correlation that might exist between the seventy-seven identified metabolites or between twenty-one study subjects (online Supplementary Fig. S2(a), (b)). Between the two cohorts, metabolites such as taurine, xanthine and hypoxanthine were positively correlated (red colour) to each other and negatively correlated (blue colour) to chenodeoxycholic acid, neopterin and glycocholic acid (online Supplementary Fig. S2(a)). Moreover, there was a high positive correlation observed between all the essential amino acids. This suggests a strong inter-relationship between these metabolites, which could be expected as these belong to the same metabolic pathway such as amino acid biosynthesis. Pearson’s correlation matrix analysis on the different cohort subjects, however, revealed no significant trends (online Supplementary Fig. S2(b)), suggesting no inter-relationship or correlation between the samples, which negates the possibility of any biases in the sample workflow.

Taken together, these multiple lines of evidence suggest that taurine, hypoxanthine, xanthine, chenodeoxycholic acid, neopterin and glycocholic acid are the most significant DEM in the sera of healthy controls *v* subjects with low B_12_ levels. Pathway enrichment analysis further confirmed that the alteration in taurine and hypoxanthine metabolic pathway is strongly associated with low B_12_ levels.

### Selection and identification of metabolite and/or metabolite ratio as biomarker

**Fig. 3.**
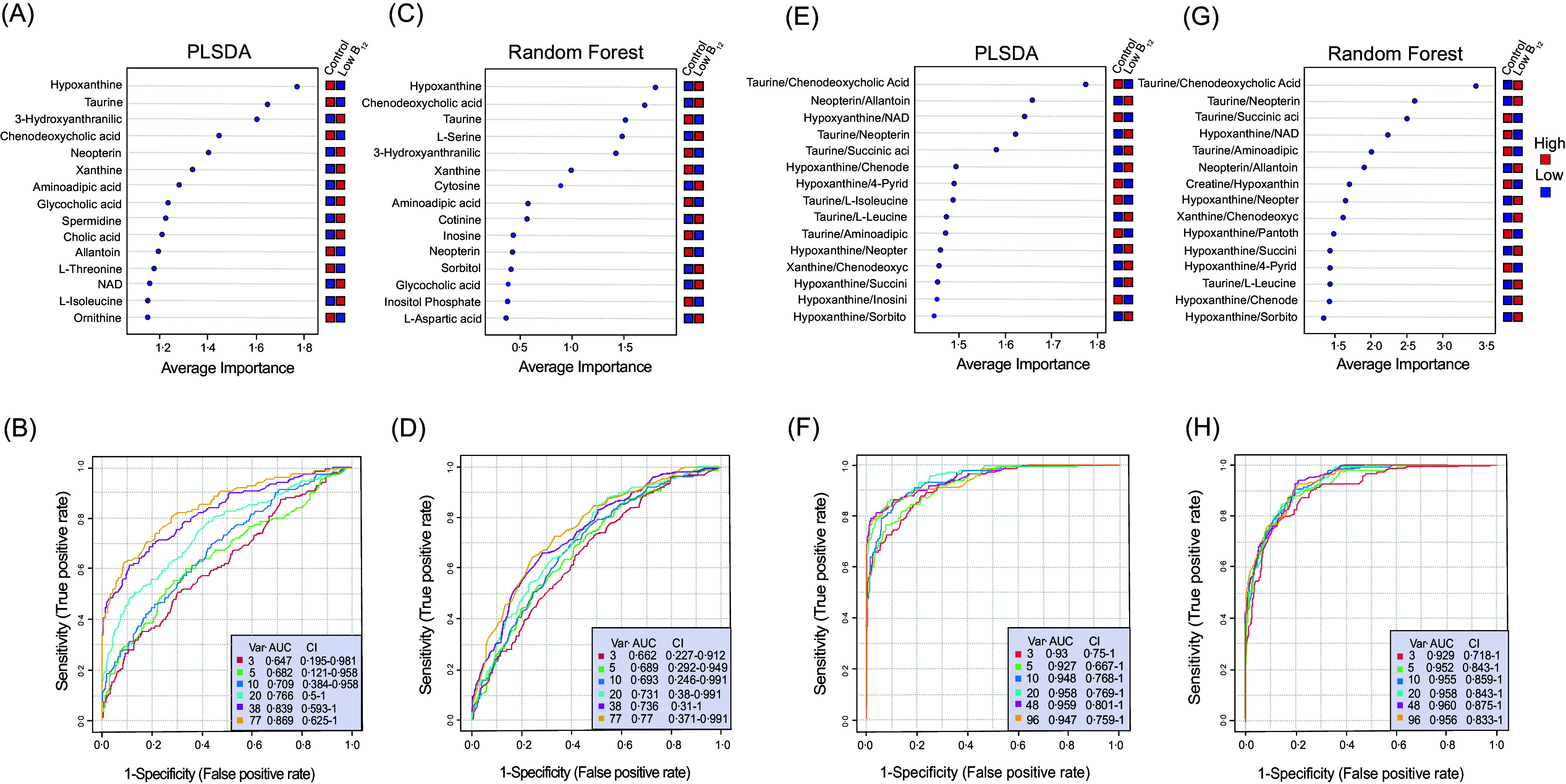
Selection and identification of metabolite and/or metabolite ratio as a biomarker. The top six predictive models (Var.) generated by various multivariant analyses were compared for their performance as metabolite biomarker predictors for low B_12_ levels using ROC–AUC curves based on the Monte-Carlo cross-validation method. ROC–AUC curve for (a) PLS-DA and (c) RF models using singular metabolites as features. ROC–AUC curve for (e) PLS-DA and (g) RF models using abundance ratio of metabolite pairs as features. Feature ranking plot for (b) PLS-DA and (d) RF models representing the top fifteen metabolites arranged in descending value of average importance score. The average importance scores range from 1 to 2 for PLS-DA and 0–2 for RF. Feature ranking plot for (f) PLS-DA and (h) RF models representing top fifteen abundance ratio of metabolite pairs arranged in descending value of average importance score. The average importance score ranges from 1 to 2 for PLS-DA and 1–4 for RF. In all the feature ranking plots, the relative abundance of each feature between the control and low B_12_ group was graded with red and blue colours representing high and low abundance, respectively.

To identify the best metabolite and/or metabolites ratio that could serve as a sensitive biomarker for the prediction of low B_12_ levels, we subjected the data to two statistical analysis tools: PLS-DA (Fig. 3(a) and (e)) and Random forest (RF) analysis (Fig. 3(c) and (g)). Multiple statistical models generated by these analyses were validated and compared for their ability to identify the metabolite or metabolites ratio which can serve as the best biomarker to predict low B_12_ state. All models generated by PLS-DA or RF were validated using receiver operating characteristic (ROC) analysis, in which Area Under the Curve (AUC) score was used to monitor the sensitivity and specificity of a model (variable) in predicting the low B_12_ state. Although both are predictive modelling tools, PLS-DA analysis has a tendency to overfit even on completely random data as compared to RF analysis. Thus, the quality of the models was further assessed using Monte-Carlo cross-validation to create ROC curve for every model generated from both PLS-DA and RF analysis. These models use a combination of the most important features to build classification models, ranging from a minimum of 2 to a maximum of 100. Since Monte-Carlo cross-validation uses defined sub-sampling, 2/3 of the samples were used to evaluate the feature importance and 1/3 of the samples were used for validation. This iterative procedure was used to calculate the performance (AUC) and CI of each model and the one with AUC closest to 1 with low variability (CI) was considered to be the best model. The software gave output in the form of ROC curves of top six models, referred to as variables, based on the CV performance. We used the most significant DEM (Fig. 3(a) and (c)) or metabolite ratio (Fig. 3(e) and (g)) as top features to generate best six models for the prediction of low B_12_ state. Note that the nomenclature of models (referred to as variables, hereinafter) is representative of the number of features used to create the model. Fig. 3(b), (d), (f) and (h) represent the ROC curve for the top six models obtained following PLS-DA and RF analysis, whereas the model numbers 1–6 represent the variables (Var.) 3 (red), 5 (green), 10 (blue), 20 (cyan), 28 (pink) and 77 (yellow), respectively, signifying that model 1 was created using two metabolites of top importance, whereas model 6 used top seventy-seven metabolites.

Both PLS-DA (Fig. 3(a)) and RF (Fig. 3(c)) analysis, using singular metabolites as features, showed that models with more than twenty metabolites (38 and 77) have high AUC (> 0·7) and tight CI, suggesting their potential to be better models, compared with those with fewer than twenty metabolites. A higher score suggests a better predictive ability of a model to identify the low B_12_ state. The feature ranking plot for both PLS-DA (Fig. 3(b)) and RF (Fig. 3(d)) analysis showed the top fifteen metabolites arranged in descending order of average importance scores contributing to the model accuracy. The average importance scores of hypoxanthine and taurine were among the top three metabolites in both analyses, with hypoxanthine having the maximum score. Both models showed lower (blue) abundance of taurine in subjects with low B_12_ levels, but the same was not true for hypoxanthine. This was consistent with PLS-DA analysis done in Fig. 2(f). It is important to note that (a) seven of fifteen top metabolites were different between the models generated by PLS-DA and RF and (b) the individual average importance score for the eight identical metabolites varied in the two analyses. This suggested that both analyses work on independent algorithms and there was no bias in the selection of hypoxanthine and taurine as top metabolite biomarkers for predicting low B_12_ state.

Next, we investigated whether abundance ratios of metabolite pairs could increase the sensitivity of PLS-DA and RF models to detect low B_12_ state (Fig. 3(c) and (d)). Ratios of all possible metabolite pairs were computed, and top-ranked ratios (based on p values) and top twenty were included for biomarker analysis. Using abundance ratios of metabolite pair as a feature, both PLS-DA (Fig. 3(e)) and RF (Fig. 3(g)) models showed that all the top six models have high AUC (> 0·9) and high CI which were comparable, suggesting any model with more than three features was a good model with high specificity and sensitively but high variability (scattered CI) as well. One-to-one comparison of AUC and CI scores for both the PLS-DA and RF models based on the abundance ratios of metabolite pair *v* singular metabolites revealed that the former can serve as better biomarkers in predicting low B_12_ state. The feature ranking plot for models in Fig. 3(f) and (h) listed thirteen identical sets of metabolite pairs with taurine/chenodeoxycholic acid gaining the highest average importance score in both (Fig. 3(g)–(h)). The abundance of taurine/chenodeoxycholic acid ratio however was reversed in the two models, being low (blue) in PLS-DA and high (red) in RF for subjects with low B_12_ levels (Fig. 3(e) and (g)). It is important to note that this analysis was consistent with the previous analysis shown in Fig. 2 (principal component analysis, volcano plot, PLS-DA and univariate analysis).

Together, results suggest that out of the metabolites identified to be differentially expressed between healthy controls and low B_12_ group taurine, hypoxanthine and the ratio of taurine/chenodeoxycholic acid could serve as biomarkers for low B_12_ levels.

### Comparison of the abilities of taurine, hypoxanthine and taurine/chenodeoxycholic acid ratio to predict low B_12_ state

**Fig. 4.**
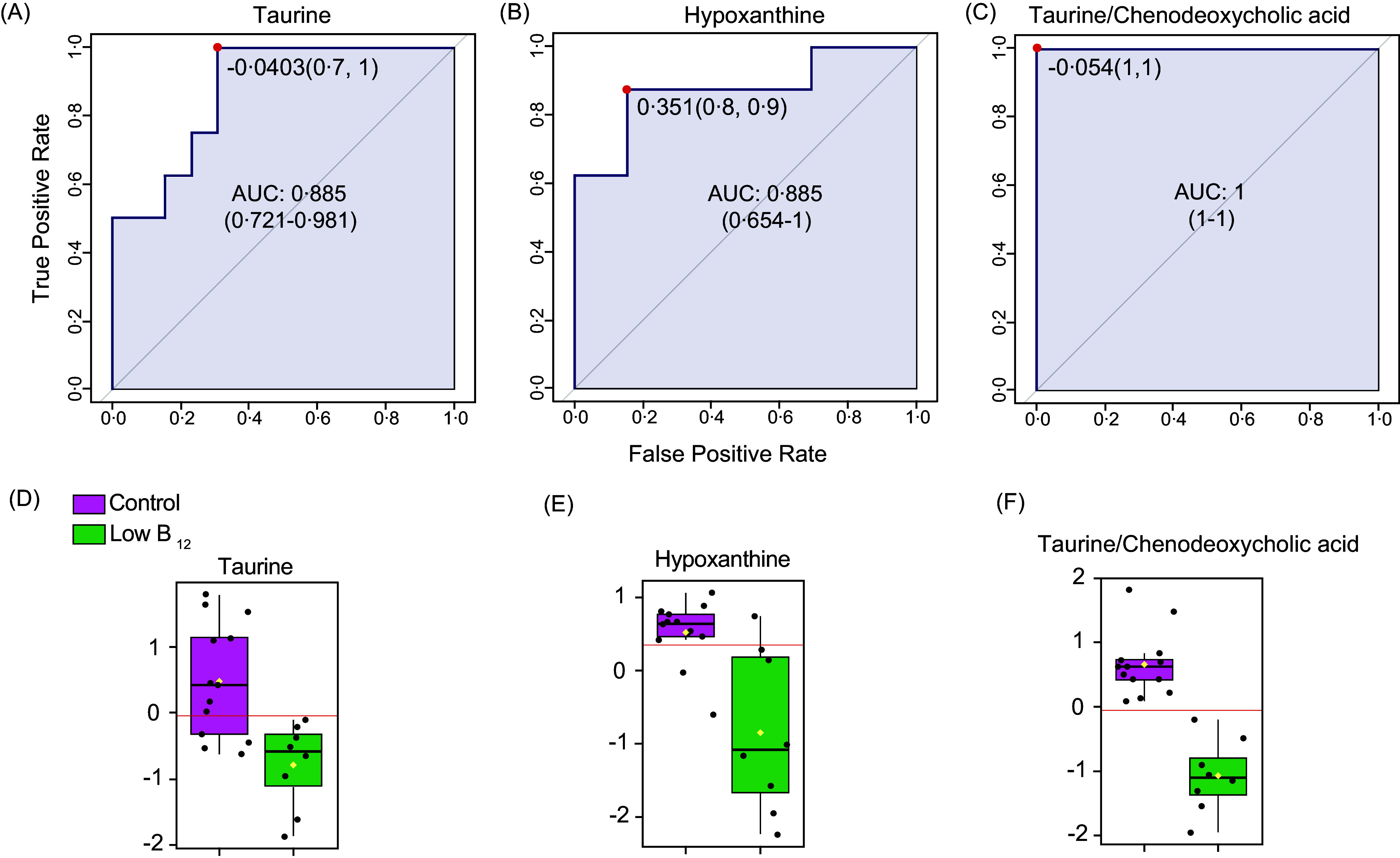
Comparison of the abilities of taurine, hypoxanthine and taurine/chenodeoxycholic acid ratio to predict low B_12_ state. ROC–AUC curve showing performance of (a) taurine, (b) hypoxanthine and (c) taurine/chenodeoxycholic acid ratio as biomarker to predict low B_12_ levels based on AUC (sensitivity, specificity) and CI (variability) values. Each ROC curve is a plot between false positive rate (x-axis) and true positive rate (y-axis). Box plots showing normalised concentration of (d) taurine (e) hypoxanthine and (f) taurine/chenodeoxycholic acid ratio between control (pink) *v* low B_12_ (green) group. Each dot represents a sample. Y-axis represents fold change values. *P* value <0·05.

We performed ROC analysis to further characterise the predictive ability of taurine alone, hypoxanthine and taurine/chenodeoxycholic acid ratio, which were shortlisted from previous PLS-DA and RF analyses. The sensitivity and significance of taurine, hypoxanthine and taurine/chenodeoxycholic acid in predicting low B_12_ state is represented using AUC score from ROC analysis (Fig. 4(a)–(c)). The scaled concentration of the indicated metabolites are shown in Fig. 4(d)–(f). This analysis showed that AUC for taurine/chenodeoxycholic abundance ratio was 1, which is equivalent to being a perfect diagnostic biomarker (Fig. 4(c)). Furthermore, the AUC and *P*-values for taurine/chenodeoxycholic acid ratio were the lowest (*P*-value=5·3193E-7) in comparison to hypoxanthine (AUC = 0·885, *P*-value =7·0513E-4) and taurine alone (AUC = 0·885, *P*-value =0·002), suggesting that taurine/chenodeoxycholic ratio was the best variable as a biomarker to predict low B_12_ state compared to others. Between taurine and hypoxanthine, the AUC scores were comparable, but hypoxanthine was significant in differentiating the two groups because of the lower *P*-value.

These results suggest that serum taurine/chenodeoxycholic acid abundance ratio can serve as a diagnostic biomarker for predicting low B_12_ state with high specificity and sensitivity.

**Fig. 5.**
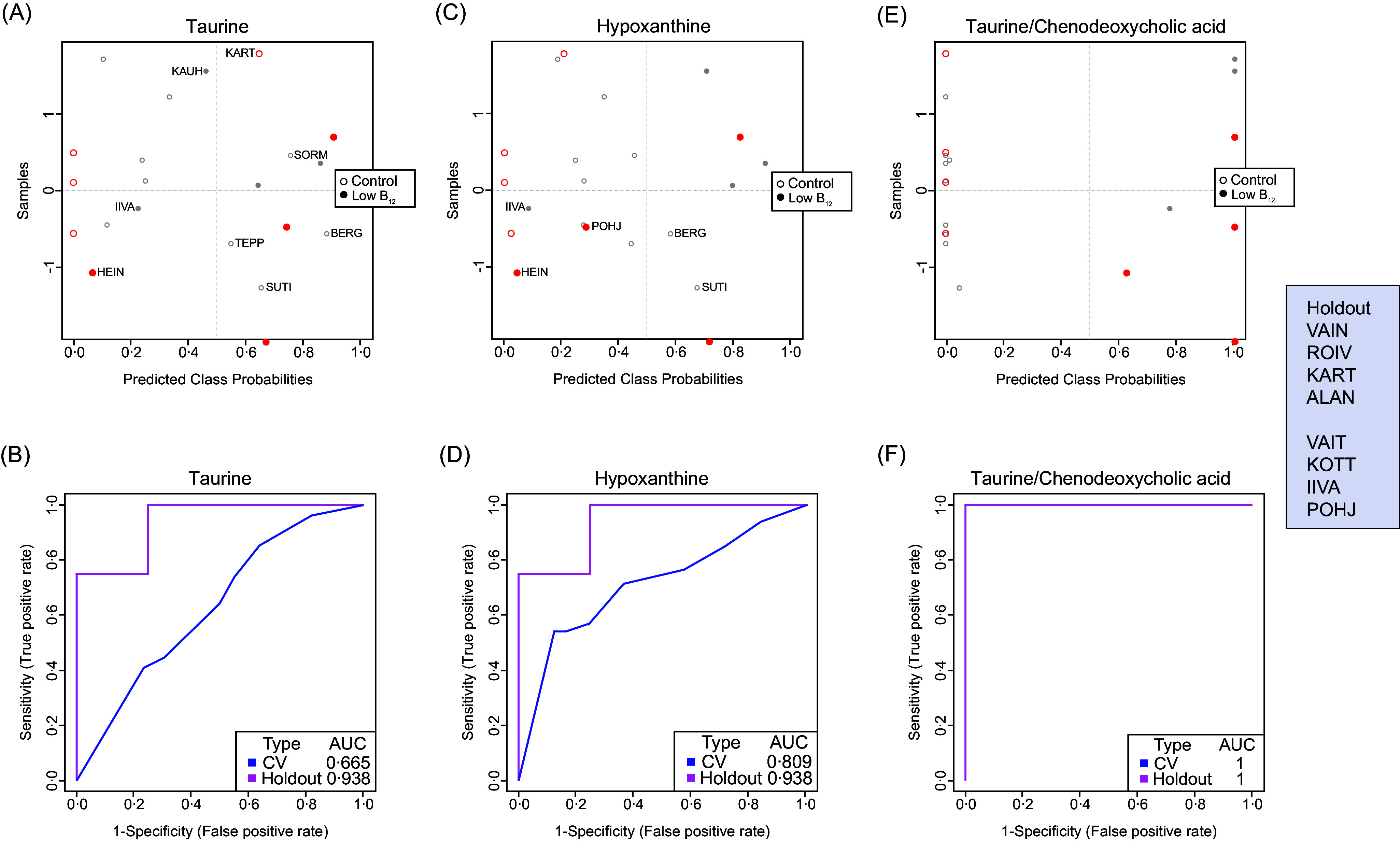
Statistical model to test predictive ability of taurine alone and in combination as biomarker. Random forest was used as a model to test the predictive abilities of taurine, taurine and hypoxanthine together and taurine/chenodeoxycholic acid ratio to predict low B_12_ levels. Predicted class probability plot for (a) taurine, (b) taurine and hypoxanthine together and (c) taurine/chenodeoxycholic acid ratio showing the classification accuracy of each factor to differentiate between control (grey dots) and low B_12_ (red dots) samples. The solid dots are training data sets, and the empty dots are test data sets. ROC–AUC curve analysis showing cross-validation (pink) and hold-out (blue) scores to determine the performance of (d) taurine, (e) taurine and hypoxanthine and (f) taurine/chenodeoxycholic acid ratio as a biomarker to predict B_12_ deficiency. Each ROC curve is a plot between the false positive rate (specificity) on the x-axis and true positive rate (sensitivity) on the y-axis.

To further test the ability of RF using taurine alone or/and in combination with other metabolites as biomarker to predict low B_12_ state, we trained a RF model on train data using cross-validation and predicted on the test data. For unbiased assessment, equal number of samples (*n* 4/group) were randomly selected from control and subjects with low B_12_ levels as hold-out samples. These samples were not used for fitting process in the model but used as testing samples. The rest of the samples were used as training samples to predict low B_12_ state. We compared predictive ability of taurine alone, taurine and hypoxanthine and ratio of taurine/chenodeoxycholic acid using AUC score (ROC analysis), predicted class probabilities and cross-validation (CV) prediction (Fig. 5). Amongst these model (Fig. 5(a), (c) and (e)) comparisons, taurine/chenodeoxycholic acid showed the highest margin of separation between the controls (empty grey circles, left edge of x-axis) and subjects with low B_12_ levels (filled grey circles, right edge of x-axis) group in training set (Fig. 5(e)). Also, the hold-out samples from both groups (control = empty red circles, low B_12_ subjects = red filled circles) fit perfectly well with the corresponding group in the testing data set. Moreover the ROC–AUC curve showed that taurine/chenodeoxycholic abundance ratio had the highest accuracy (AUC CV = 1, AUC holdout = 1, Fig. 5(f)) in predicting low B_12_ state compared to taurine alone (AUC CV = 0·665, AUC holdout=0·938, Fig. 5(b)) or hypoxanthine (AUC CV = 0·809, holdout = 0·938, Fig. 5(d)). Overall, this analysis was consistent with previous RF analysis, suggesting towards great potential of taurine/chenodeoxycholic acid to serve as serum biomarker for predicting low B_12_ state.

### Metabo-transcriptomic network analysis linked B_12_-dependent reactions with taurine/chenodeoxycholic acid

We performed a network analysis of differentially expressed genes and metabolites between controls and B_12_-deficient livers in a mouse model of B_12_ deficiency reported previously by us^(30)^. Liver is a suitable tissue to investigate effects of B_12_ deficiency since it is one of the principal site of B_12_ storage, and we demonstrated earlier that B_12_ deficiency compromises its functions^(30)^. In the cells, B_12_ is known thus far to be converted into two cofactors (methyl-B_12_ and adenosyl-B_12_), which are required for the functioning of two known enzymes, methionine synthase and methyl-malonyl CoA mutase^(31,32)^. Thus, we focussed our attention on metabolic pathways that are interconnected with the B_12_-derived cofactor-dependent reactions such as Krebs cycle, amino acid metabolism, urea cycle and nucleotide metabolism.

**Fig. 6.**
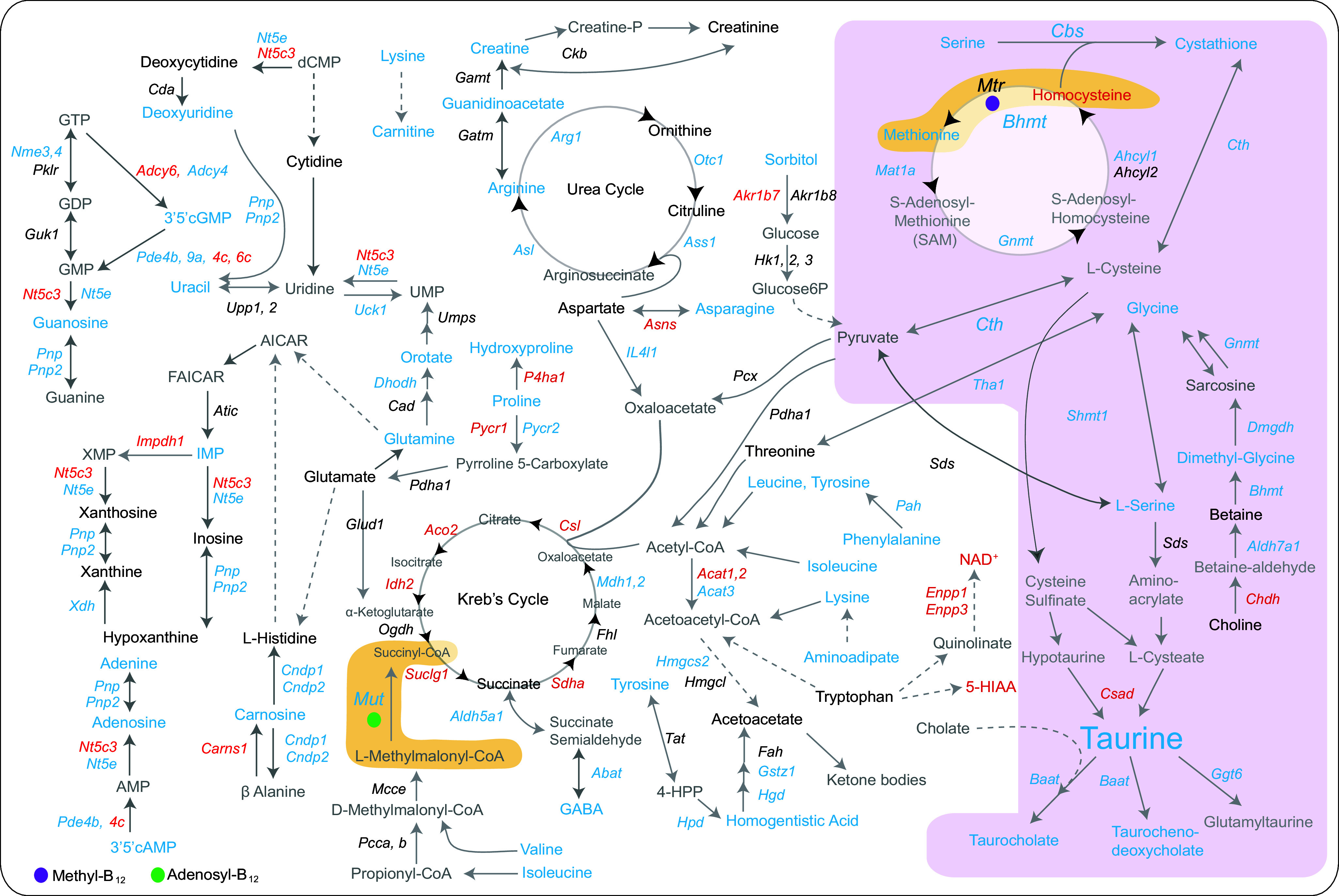
Metabo-transcriptomic network analysis links B_12_-dependent reactions with taurine/chenodeoxycholic acid. Network analysis showing the differentially expressed genes and metabolites between controls and B_12_-deficient livers in a mouse model of B_12_ deficiency reported previously^(27)^. The network shows interactions between enzymes (italics font) and metabolites (normal font) across various metabolic pathways in the liver, such as Krebs cycle, urea cycle, amino acid metabolism, nucleotide metabolism, etc. The arrows represent the direction of the reaction. The downregulation and upregulation of enzyme transcript or metabolite concentrations are represented by blue and red colour, respectively. Black represents no change, while grey represents not measured.

The network visualisation of differentially expressed transcriptome showed that transcripts encoding the enzymes that catalyse metabolite conversions in these pathways were overall downregulated (in blue), except for the Krebs cycle, in which expression of five out of nine enzymes was upregulated (in red) (Fig. 6). This upregulation in the expression levels of Krebs cycle enzymes could be linked to decreased activity of methyl-malonyl CoA mutase (Mut), which is dependent on the adenosyl-B_12_ for its activity. Mut catalyses the synthesis of Succinyl-CoA, an intermediate in the Krebs cycle that plays a critical role in providing protons for the OXPHOS system, and thus, energy production in the cells. B_12_ deficiency leads to an energy deficit in the cells, and consequently likely, a compensatory increase in the expression levels of enzymes in the Krebs cycle. However, no reactions surrounding the adenosyl-B_12_-dependent Mut enzyme and Krebs cycle could relate to known taurine biosynthetic machinery in B_12_-deficient cells.

An analysis of reactions surrounding methionine synthase (Mtr), the second enzyme that is dependent on the methyl-B_12_ as a cofactor, showed that the concentrations of methionine, the downstream product, were decreased while concentrations of its precursor, homocysteine, were increased (Fig. 6). Expression levels of the enzymes in the methionine cycle were either not affected or were decreased. The methionine cycle is linked to cysteine synthesis through transsulfuration pathway in the cells and through a series of reactions, to taurine biosynthesis. Most of the enzymes and their downstream products in this pathway were downregulated, consequently leading to deficiency of multiple metabolites in taurine metabolic pathway (taurine, taurocholate, tauro-chenodeoxycholate) (Fig. 6). The expression levels of the enzyme, *Csad*, that catalyses the rate limiting step in taurine biosynthesis, was increased likely as a compensatory mechanism due to deficiency of taurine (Fig. 6).

Further analysis of gene-metabolite networks interconnected with B_12_-dependent reactions showed that gene expression of enzymes and metabolite intermediates in the urea cycle were downregulated. In the amino acid metabolism pathways, barring tryptophan metabolite, HIAA and NAD^+^ pathways, all enzyme expressions and metabolite intermediates were downregulated. In the nucleotide metabolism pathways, metabolite intermediates were either downregulated or not affected, and apart from a few enzymes, most of the enzyme expressions were downregulated.

Together, these integrated metabolomic and transcriptomic analyses in the WT and B_12_-deficient liver samples revealed global downregulation of metabolic networks upon B_12_ deficiency and identified a hitherto unanticipated connectivity between B_12_-dependent reactions and taurine metabolism.

## Discussion

By using metabolomic analysis of serum from controls and subjects with low B_12_ levels, we were able to identify that a ratio of taurine/chenodeoxycholic acid levels can serve as a biomarker of, difficult to detect, low B_12_ state. The quantitative metabolomic analysis of seventy-seven relevant metabolites in the sera of subjects with low B_12_ levels revealed that most of the metabolites were downregulated and are involved in metabolism of amino acids, betaine, glutathione, bile acid and purines (Fig. 2). Metabolite set enrichment analysis on the perturbed metabolite profiles showed alterations in the metabolic pathways associated with amino acid and methionine metabolism (Fig. 1). Downregulation in methionine levels in this metabolome is consistent with the role of B_12_ as an essential cofactor of methionine synthase, while homocysteine accumulated from the dysfunction of methionine synthase was 1·8-fold elevated. Furthermore, univariate analysis of the metabolome from subjects with low B_12_ levels identified a differential abundance of taurine, hypoxanthine and xanthine between the two groups. The multivariate RF analysis aimed towards identifying which metabolite(s) contributed to the separation of the two groups with higher specificity and sensitivity showed taurine/chenodeoxycholic ratio as the metabolic parameter that could separate the two groups with 99 % accuracy. Thus, we propose taurine/chenodeoxycholic acid ratio as a potential biomarker of a low B_12_ state in humans.

Previous studies have characterised the human serum metabolome in B_12_-deficient subjects in an attempt to reveal connections between B_12_-deficient state and serum metabolic markers. Brito *et al.* performed metabolomic profiles in sera of Chilean older adults with subclinical borderline B_12_ deficiency following an intervention with a single injection of commercial vitamin B_12_ (10 mg) along with pyridoxine (100 mg) and thiamine (100 mg) (defined by serum B_12_ < 148 pmol/l, holotranscobalamin <35 pmol/l, tHcy >15 μmol/l, or methylmalonic acid > 271 nmol/l)^(33)^. Although this study showed perturbations in multiple metabolite such as acylcarnitine and plasmalogens, the authors did not have a vitamin B_12_ alone arm in their studies and did not subject their data to downstream algorithms to identify potential biomarkers of B_12_ levels. Moreover, the previous study did not include a control B_12_ intervention group. Although these studies provide evidence that serum metabolome could be altered by low B_12_ state, it was unknown whether any of the metabolites or set of metabolites could serve as a biomarker of B_12_-deficient state. Our study fills this gap in our knowledge and elucidates the effect of low B_12_ levels specifically on the cellular, metabolic and transcriptomic landscape of the cell using liver biopsies from a B_12_-deficient mouse model. Together, these studies pave a way towards better understanding of the cellular defects caused by low B_12_ levels.

We acknowledge that our study has certain limitations. First, the small sample size limits the statistical power of the RF models. Repeating the same study in a larger sample size may allow a greater number of metabolites to pass quality control for downstream analysis. Second, the current study population was only tested for B_12_ levels and not for the consequences of B_12_ deficiencies such as neurological or haematological abnormalities. Third, samples used in our study are a subset of KIHD study, which assessed the effect of dietary changes on cardiovascular risk. We cannot identify/be certain of the reason for low B_12_ levels or the presence of other molecular deficiencies. Therefore, changes in serum B_12_ levels in the two groups we studied could be either due to reduced dietary intake, absorption or increased metabolism of B_12_. Future studies thus need to investigate whether reduced intake/absorption *v* metabolism of B_12_ affects the metabolite levels differentially in the sera of subjects with low B_12_ levels. Fourth, present study is a cross-sectional study, and a cause-and-effect relationship cannot be determined. A B_12_ *v* placebo intervention trial will therefore be necessary in the future to see if the taurine/chenodeoxycholic acid ratio will respond to B_12_ supplements. These and other questions will need to be addressed in future studies.

Vitamin B_12_ deficiency leads to perturbed levels of taurine, hypoxanthine, xanthine, chenodeoxycholic acid, neopterin and glycocholic acid. We show that taurine levels alone and taurine/chenodeoxycholic acid ratio are promising candidates for serum metabolite-based biomarkers to identify low B_12_ state. The two critical metabolites identified in this study affected by B_12_, taurine and chenodeoxycholic acid, belong to the taurine metabolic pathway. Taurine metabolism gets compromised with age and leads to taurine deficiency in humans; however, the cause of this deficiency is unknown^(26)^. The present study identifies vitamin B_12_ as the very first upstream effector of taurine metabolism in humans and illustrates the transcriptomic and metabolomic changes through which B_12_ could affect this process. Our results of low taurine abundance in the sera of low B_12_ subjects are consistent with the previous reports that have shown increased excretion of taurine and may imply, albeit indirectly, lower taurine in the blood, in pernicious anaemia patients^(34–36)^. These later indirect evidences are further supported by the demonstration of >90 % decreased in taurine abundance in the blood of a mouse genetic model of *Gif*-deficiency^(27)^. These results are significant given that taurine deficiency has recently been shown to be a driver of ageing in diverse species and is associated with poor health in humans. This study paves a way for future clinical work to streamline diagnostic tools to detect low B_12_ state through a simple blood test and perhaps other age-associated diseases.

## Material and methods

### Chemicals and reagents

All the metabolite standards, ammonium formate, ammonium acetate and ammonium hydroxide were obtained from Sigma-Aldrich (Helsinki, Finland). Formic acid (FA), 2-proponol, acetonitrile (ACN) and methanol (all HiPerSolv CHROMANORM, HPLC grade, BDH Prolabo) were purchased from VWR International (Helsinki, Finland). Isotopically labelled internal standards were obtained from Cambridge Isotope Laboratory. Inc., USA (Ordered from Euriso-Top, France). Deionised Milli-Q water up to a resistivity of 18 MΩ cm was purified with a purification system (Barnstead EASYpure RoDi ultrapure water purification system, Thermo scientific, Ohio, USA).

### Metabolite extraction protocol

The working calibration solutions were prepared in ninety-six-well plates by serial dilution of the stock calibration mix using Hamilton’s MICROLAB® STAR line (Hamilton, Bonaduz AG, Switzerland) liquid handling robot system. Starting from a stock solution mix, ten additional lower-working solutions were prepared using water as the diluent to build the calibration curves.

### Clinical serum samples

Clinical samples used for assessing the changes in vitamin B_12_ levels and metabolites in blood are from the KIHD study, a population-based cohort study described previously^(27,37)^ and were donated by J. Kauhanen and T. Nurmi (University of Eastern Finland, Kuopio, Finland). KIHD study was conducted according to the guidelines laid down in the Declaration of Helsinki, and all procedures involving human subjects were approved by the University of Eastern Finland (details are included in the original publication reference 37). Written informed consent was obtained from all subjects in the KIHD study. Briefly, KIHD study was a prospective, population-based cohort study in eastern Finland population aimed to determine associations between dietary factors and risk of major chronic diseases such as cardiovascular diseases. KIHD study focused on the effect of diet on cardiovascular health specifically. Consumption of foods was assessed at baseline in 1984–1989 with the use of a 4-d guided food recording by household measures, 1 day of which was a weekend day. A picture book of 126 most common foods and drinks consumed in Finland (including proteins, SFA, MUFA, PUFA, carbohydrates, eggs, processed red meat, fruits, berries and vegetables) was used to help in the estimation of portion sizes. The intake of nutrients was estimated with the use of NUTRICA version 2·5 software (Social Insurance Institution, Finland). The outcome assessment for the study included chronic conditions such as cardiovascular diseases, carotid atherosclerosis, diabetes, metabolic syndrome, cancer, inflammatory disease, cognitive decline, liver disease, death, pain, chronic depression and infection. Ten microliters of labelled internal standard mixture was added to 100 μl of serum sample. Metabolites were extracted by adding four parts (1:4, sample: extraction solvent) of the 100 % ACN + 1 % FA solvent. The collected extracts were dispensed in OstroTM 96-well plate (Waters Corporation, Milford, USA) and filtered by applying vacuum at a delta pressure of 300–400 mbar for 2·5 min on robot’s vacuum station. This resulted in a cleaner extract to the 96-well collection plate, which was placed under the OstroTM plate. The collection plate was sealed with the cap map and placed in an auto-sampler of the LC system for the injection.

### Instrumentation and analytical conditions

Sample analysis was performed on an ACQUITY UPLC-MS/MS system (Waters Corporation, Milford, MA, USA). The auto-sampler was set at 5°C, and the column, 2·1 × 100 mm Acquity 1·7 um BEH amide HILIC column (Waters Corporation, Milford, MA, USA), temperature was maintained at 45°C. The total run time is 14·5 min including 2·5 min of equilibration step at a flow rate of 600 μl/min. Initially, the gradient started with a 2·5 min isocratic step at 100 % mobile phase B (ACN/ H2O, 90/10 (v/v), 20 mM ammonium formate, pH at 3) and then rising to 100 % mobile phase A (ACN/H2O, 50/50 (v/v), ammonium formate, pH at 3) over the next 10 min and maintained for 2min at 100 % A and finally equilibrated to the initial conditions for 2·5 min. An injection volume of 5 μl of sample extract was used, and two cycles of 300 μl of strong wash (methanol/isopropanol/ACN/H2O, 25/25/25/25, 0·5 % FA) and 900 μl of weak wash (methanol/isopropanol/ACN/H2O, 25/25/25/25, 0·5 % ammonium hydroxide) and in addition 2 min of seal wash (90/10, methanol/H2O) were carried out. The auto-sampler was used to perform a partial loop with needle overfill injections for the samples and standards.

The detection system, a Xevo® TQ-S tandem triple quadrupole mass spectrometer (Waters, Milford, MA, USA), was operated in both positive and negative polarities with a polarity switching time of 20 msec. Electrospray ionisation was chosen as the ionisation mode with a capillary voltage at 0·6 KV in both polarities. The source temperature and desolvation temperature of 120°C and 650°C, respectively, were maintained constantly throughout the experiment. Declustering potential and collision energy were optimised for each compound. High pure nitrogen and argon gas were used as desolvation gas (1000 l/hr) and collision gas (0·15 ml/min), respectively. Multiple reaction monitoring acquisition mode was selected for quantification of metabolites with individual span time of 0·1 sec given in their individual multiple reaction monitoring channels. The dwell time was calculated automatically by the software based on the region of the retention time window, number of multiple reaction monitoring functions and depending on the number of data points required to form the peak. MassLynx 4·1 software was used for data acquisition, data handling and instrument control. Data processing was done using TargetLynx software, and metabolites were quantified by using labelled internal standards and external calibration curves.

### Data analysis using MetaboAnalyst 5·0 software and downstream analysis

The raw data was analysed using MetaboAnalyst 5·0 software (https://www.metaboanalyst.ca/)^(38,39)^. Metabolite raw values were generalised log (glog) transformed and auto-scaled (mean-centered and divided by the standard deviation of each variable)^(40)^. Missing values for any metabolites in the sample below the limit of detection were inputted with 1/5 of the minimum positive value for each variable. Unsupervised principal component analysis was done to differentially cluster the two groups^(41,42)^. Hierarchical clustering and Pearson’s correlation analysis were also performed to cluster the metabolite and sample data in the form of a heatmap to easily identify patterns in metabolite concentrations across samples. Metabolite set enrichment analysis^(43)^ was performed on all metabolites with a variable importance projection ≥ 1·5 that matched the database using the ‘Pathway-associated metabolite sets (SMPDB)’ database in the MetaboAnalyst software. Pathway analysis was performed using the ‘Homo sapiens (KEGG^(44,45)^)’ database in the MetaboAnalyst software. Interactive scatter plot with ‘Enrichment Factor’ as *x* axis and ‘−log_10_(*P*)’ as *y* axis was generated for functional analysis to show the significance of top fifty metabolic pathways involving the metabolites identified. The variable importance to projection score for each metabolite was calculated to quantitatively represent metabolite feature importance in the model. A volcano plot scatterplot that shows statistical significance (–log10(*P*-value) *v* magnitude of change (log 2-fold change) of metabolites. Metabolites that show significant (*P* ≤ 0·05) change (log 2-fold change ±0·5) are highlighted. Multivariate supervised PLS-DA and RF analysis were performed to assess the difference between the abundance of top metabolites or metabolite ratio between the two groups. The AUC of the ROC curve was also calculated for each metabolite to determine its predictive ability as a biomarker. The ROC curve is a plot of false positive rate *v* the true positive rate. The higher the AUC value, the better the measurements are at classifying between the two groups.

### Mouse studies

Mouse liver data used for the generation of metabolite and transcriptomic networks was obtained from our previously published study^(27)^. All procedures performed on mice conformed to the ethical regulation guidelines of the Wellcome Trust Sanger Institute and to the guidelines of the UK Home Office (project license no. PPL80/2479). Brief description of the procedures is described below. Frozen liver samples (*n* 5, each group) were collected from control and B_12_ deficient mice (C57Bl/6N strain, females). All animals were housed at a pathogen-free facility at the Sanger Institute animal facility and had free access to food and water. No randomisation was performed during tissue collections. No experimental procedures were performed on the animals, and tissues were obtained after euthanasia in a CO_2_ chamber. Samples were weighed (20–40 mg) and transferred to precellys homogenisation tubes (Precellys 24 lysing kit, precellys) containing 1·4 mm ceramic (Zirconium oxide) beads by adding 10 μl of labelled internal standard mix and incubated on ice for 10 min. After incubation, twenty parts of extraction solvent was added to the sample (1:20, sample:extraction solvent). In order to gain maximum recovery of small molecules, the homogenisations were performed with a Precellys 24 homogenizer (Precellys, Finland) in a two-step extraction process. In the first step, ten parts of precooled 100 % ACN + 1 % FA were added to the sample and homogenised for three cycles of 20 sec each at 5500 rpm with 30 sec pause between each homogenisation interval. After homogenisation, the sample tubes were centrifuged for 2 min at 5000 rpm at –2° C in an Eppendorf 5404R centrifuge, and the supernatant was collected in a 1·5-ml eppendorf tube. In the second step, ten parts of 80/20 % ACN/H2O + 1 % FA were added to the remaining pellet and repeated the steps as above and finally pooled to the previous extract. Instrument and analytical conditions were identical to that of the human serum sample analysis. Liver samples were processed for RNA expression analysis as described previously by us^(27)^. RNA was extracted from control and B_12_-deficient liver samples and processed for RNA sequencing analysis. Expression levels of genes that code for protein products that regulate metabolite conversions were extracted from the RNA sequencing data. Genes showing significant (*P* < 0·05) changes in expression have been presented in red and green colour; genes that do not show a change have been shown in black.

## Supporting information

Baghel et al. supplementary material 1 Baghel et al. supplementary material

Baghel et al. supplementary material 2 Baghel et al. supplementary material

## Supplementary material

For supplementary material/s referred to in this article, please visit https://doi.org/10.1017/S0007114524002022
