# Supplementary figures and images for: Taurine/chenodeoxycholic acid ratio as a potential serum biomarker for low vitamin B12 levels in humans
Source: Br J Nutr. 2024 Oct 4;132(6):712–24. doi: 10.1017/S0007114524002022 (PMC11557292; doi:10.1017/S0007114524002022)

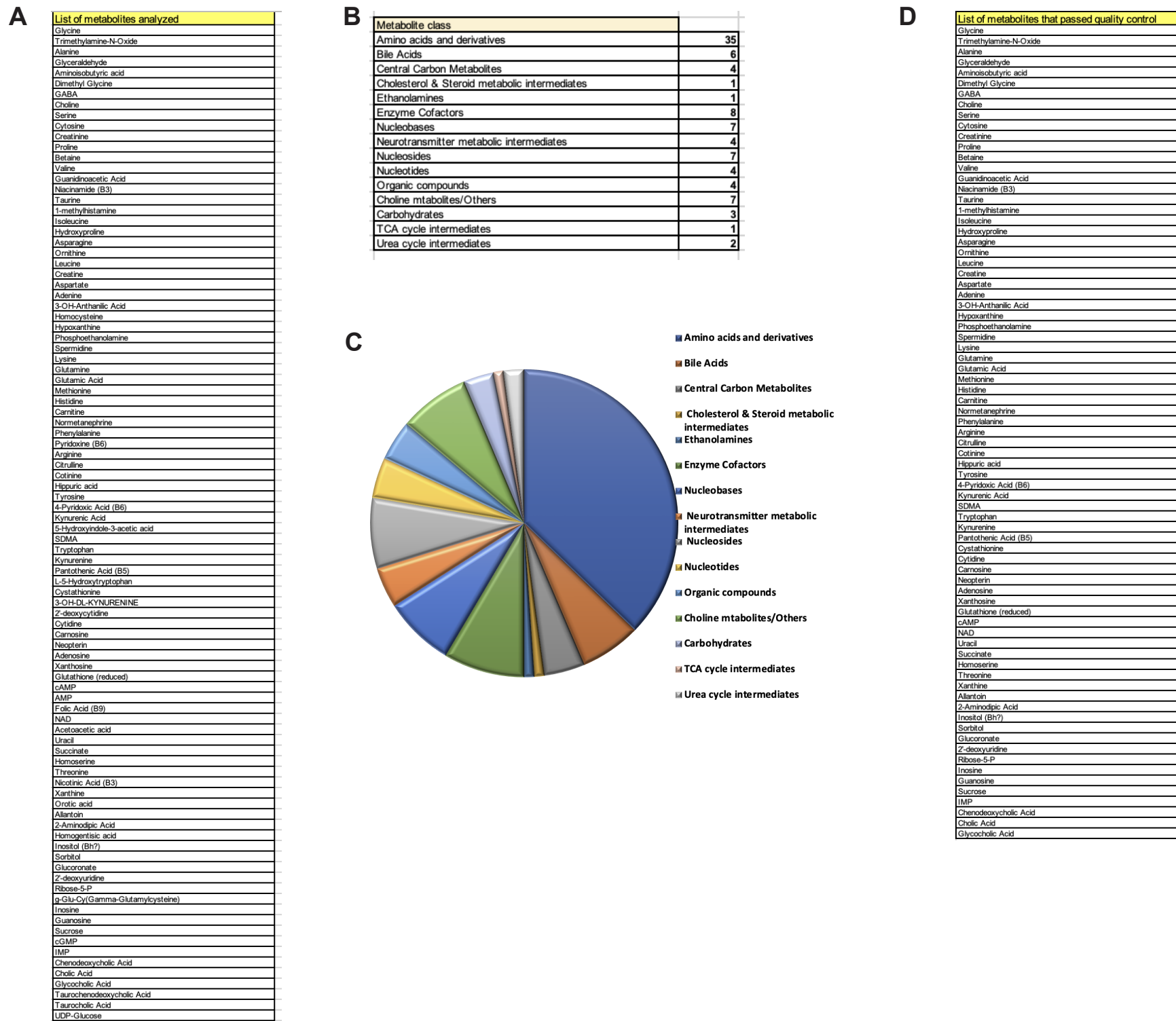

**Figure S1**

Supplement: Baghel et al. supplementary material 1 — Baghel et al. supplementary material [file S0007114524002022sup001.pdf]

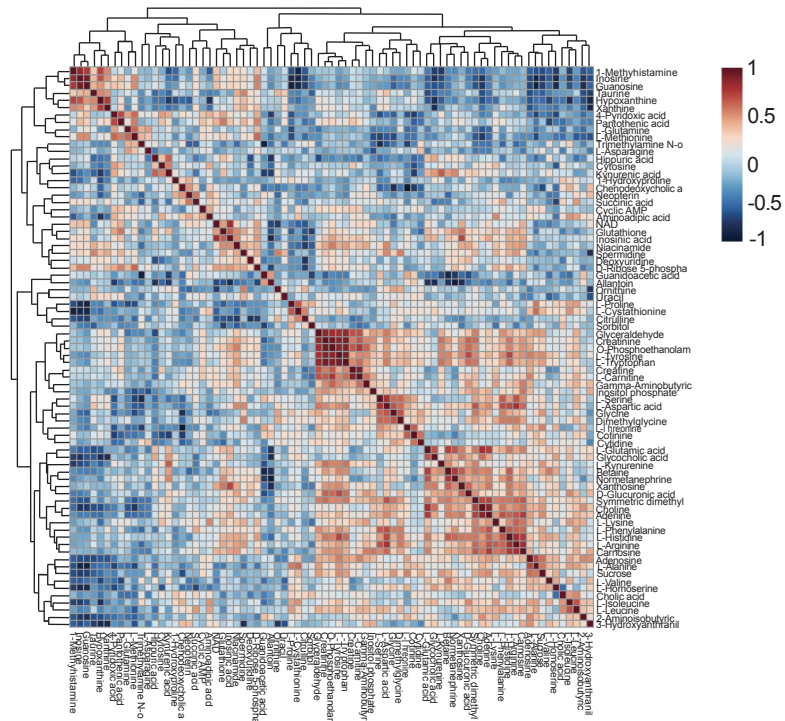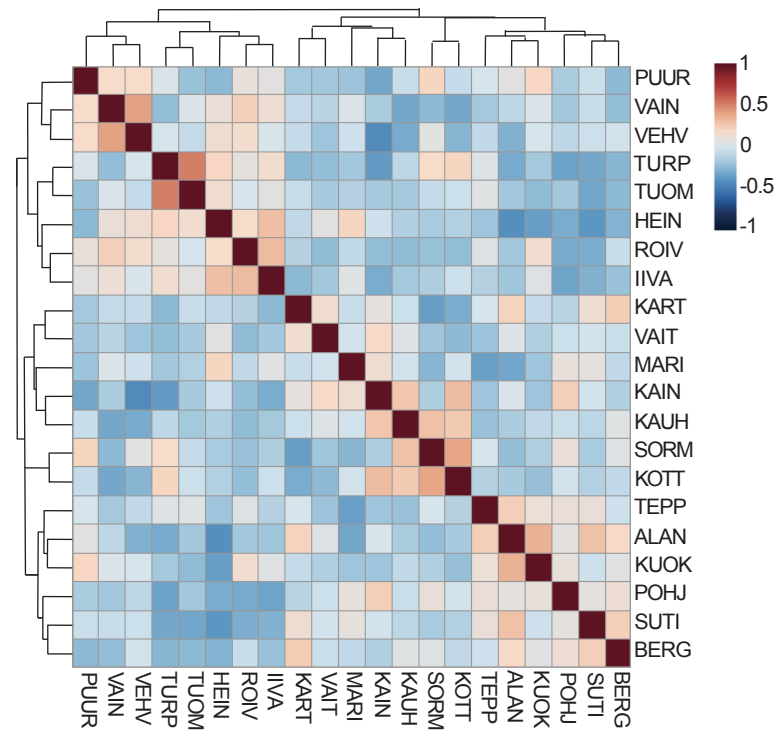

### Figure S2

Supplement: Baghel et al. supplementary material 2 — Baghel et al. supplementary material [file S0007114524002022sup002.pdf]
